# TRAJECTORIES OF REAL-WORLD VERSUS PERCEIVED MOBILITY AND LOCOMOTOR CAPACITY IN OLDER FRACTURE PATIENTS

**DOI:** 10.1093/geroni/igae098.0741

**Published:** 2024-12-31

**Authors:** Carl-Philipp Jansen, Jochen Klenk, Beatrix Vereijken, Hubert Blain, Jorunn Helbostad, Clemens Becker

**Affiliations:** Heidelberg University, Heidelberg, Baden-Wurttemberg, Germany; Robert-Bosch-Hospital Stuttgart, Stuttgart, Baden-Wurttemberg, Germany; Norwegian University of Science and Technology, Trondheim, Nord-Trondelag, Norway; Centre Hospitalier Universitaire de Montpellier, Montpellier, Aquitaine, France; Norwegian University of Science and Technology, Trondheim, Nord-Trondelag, Norway; Robert-Bosch-Hospital Stuttgart, Stuttgart, Baden-Wurttemberg, Germany

## Abstract

Hip fracture is the most frequent non-intentional injury of older persons leading to hospital admission in North America and Europe. Hip fracture rehabilitation follows the overall goal to get older persons back on their feet and mobile again, up to the point of functional and mobility-related independence in everyday life. This primary outcome, however, is measured in various forms using different methods and assessments. Oftentimes, a singular view is taken, mainly based on repeated physical and locomotor capacity measures without knowledge of the actual mobility behaviour of patients after hospital care and in real life. Cross-sectional data has shown that the relationship between perceived mobility, locomotor capacity, and real-world mobility is not straightforward, however, it remains unclear how all three aspects evolve over time. For a better understanding of this relationship in hip fracture patients, this talk will address respective data of n=566 participants of the hip fracture cohort in the Mobilise-D project. All three aspects of mobility were assessed at baseline and after 12 months. Based on the results, it will be made clear which aspects should be prioritized in both research and clinical practice to obtain a comprehensive picture and clinically useful understanding of mobility of older hip fracture patients.

